# Impact of Parkinson’s Disease on Functional Mobility at Different Stages

**DOI:** 10.3389/fnagi.2022.935841

**Published:** 2022-06-15

**Authors:** Sara Mollà-Casanova, Jose Pedrero-Sánchez, Marta Inglés, Juan López-Pascual, Elena Muñoz-Gómez, Marta Aguilar-Rodríguez, Nuria Sempere-Rubio, Pilar Serra-Añó

**Affiliations:** ^1^UBIC, Department of Physiotherapy, Faculty of Physiotherapy, Universitat de Valéncia, Valencia, Spain; ^2^Instituto de Biomecánica de Valencia, Universidad Politécnica de Valencia, Valencia, Spain

**Keywords:** Parkinson’s disease, functional assessment, FallSkip, severity of Parkinson’s, Hoen and Yahr stages

## Abstract

**Introduction:**

Specific functional assessments to determine the progression of Parkinson’s Disease (PD) are important to slow down such progression and better plan rehabilitation. This study aimed to explore possible differences in the performance of different functional tasks included in a mobility test using sensors embedded in an Android device, in people at different PD stages.

**Materials and Methods:**

Eighty-seven participants with PD agreed to participate in this cross-sectional study. They were assessed once using an inertial sensor and variables related to functional status were recorded (i.e., MLDisp, APDisp, DispA, Vrange, MLRange, PTurnSit, PStand, TTime, and RTime).

**Results:**

There was significant impairment of the vertical range during gait between stages I and II. Further, when stages II and III were compared, the sit-to-stand power was significantly impaired, and the total time required to complete the test increased significantly (*p* < 0.05). Even more significant differences were obtained when stages I and III were compared, in particular, dysfunction in postural control, vertical range, sit to stand power and total time. Finally, there were no significant differences between stages in the medial-lateral displacements and reaction time (*p* > 0.05).

**Conclusion:**

Functional mobility becomes more significantly impaired in the PD population as the PD stages progress. This implies impaired postural control, decreased ability to sit down or stand up from a chair, increased metabolic cost during walking, and overall slowing-down of motor function.

## Introduction

Parkinson’s disease (PD) is one of the most common progressive neurodegenerative diseases (Parkinson, 2002; Ascherio and Schwarzschild, 2016) whose prevalence is 1% in people over 60 years of age and 4% in individuals over 85 (Ascherio and Schwarzschild, 2016; Simon et al., 2020). It consists of the loss of dopaminergic neurons in the substantia nigra located in the midbrain and associated with Lewy bodies (Poewe et al., 2017; Simon et al., 2020). As a result, people with PD may present musculoskeletal and orthopedic problems, that produce kinesiophobia, fear of falling, and, consequently, a decrease in physical activity and daily life activities (Navarro-Flores et al., 2022). However, neurological deterioration begins years before a diagnosis can be made and has a broad range of symptoms (Poewe et al., 2017).

Because of the chronic and neurodegenerative nature of this pathology, prevention is one of the most important aspects of rehabilitation, especially fall prevention (Pickering et al., 2007; Sherrington et al., 2017). To enforce prevention in clinical care, it is important to understand how the disease behaves and how it progresses. In this way, the rehabilitation program design would be adapted to each of the PD stages (Marras et al., 2002).

Accordingly, specific assessments to determine PD progression are essential (Marras et al., 2002). Gait analysis has been proved effective to establish PD progression according to recent studies (Godi et al., 2021; Varrecchia et al., 2021; Vila et al., 2021), which have identified several spatiotemporal and kinematic parameters capable of differentiating PD stages. However, functional ability includes other motor skills indicators of static balance, such as sitting down, getting up, or turning around, and the risk of falling in this population (Pelicioni et al., 2019; Muñoz-Bermejo et al., 2021).

Previous studies have used different clinical functional tests to assess functional deterioration in PD throughout its stages, such as the Continuous Scale-Physical Functional Performance, Functional Reach Test, Timed Up and Go 360° Turn test, 6- or 2-Min Walk Test, and posture changes, among others (Martinez-Martin et al., 2006; Schenkman et al., 2011; da Silva et al., 2017). However, these studies, although showing the existence of PD progression, were unable to distinguish between consecutive stages of severity. Efforts have also been made in this regard using portable sensors (Coste et al., 2014; Iluz et al., 2014; Weiss et al., 2014; Ayena et al., 2016). However, to date, none of the studies addressing functional status in PD succeeded in differentiating between stages.

Therefore, this study aimed to explore possible differences in the performance of different functional tasks included in a mobility test using sensors embedded in an Android device, in patients with PD at varying stages.

## Materials and Methods

### Participants and Study Design

Eighty-seven participants with PD agreed to participate in this cross-sectional study. They were recruited from various PD associations [i.e., *Amigos contra el Parkinson* (València, Spain), *Asociación de Parkinson de Alicante* (Alacant, Spain) and *Asociación de Parkinson de Elche* (Alacant, Spain)].

All volunteers were following their usual rehabilitation program, customized to their needs in each association. The assessment period lasted from October 2021 to February 2022. The inclusion criteria for participation in the study were as follows: (i) PD diagnosed by a neurologist [Hoen and Yahr (HY) I, II and III] (Hoehn and Yahr, 1967), (ii) optimized and stable pharmacological therapy for at least 1 month before enrolment, and (iii) good cognitive condition defined as scores above 23 on the Mini-Mental State Examination (Folstein et al., 1975). The exclusion criteria were as follows: (i) medical contraindication of physical activity, (ii) neurological or orthopedic impairments limiting independent gait and sitting down or getting up from a chair, (iii) deafness or hearing problems, (iv) vestibular impairment, (v) blindness or vision problems, (vi) psychotic disorders, and (vii) surgical intervention in the last 6 months. All procedures were conducted in agreement with the World Medical Association Declaration of Helsinki principles. Ethical approval for the study was granted by the Ethics Committee of *Universitat de València* (H1517239006520) and all volunteers that participated in the study provided written informed consent.

### Balance, Gait, and Fall Risk Assessment

Three researchers conducted all the assessment sessions at PD association centers or the volunteer’s home. Firstly, participants were briefed on the objectives of the study and were asked to follow the researcher’s instructions. Further, training was provided to ensure the correct performance of the test.

**FIGURE 1:**
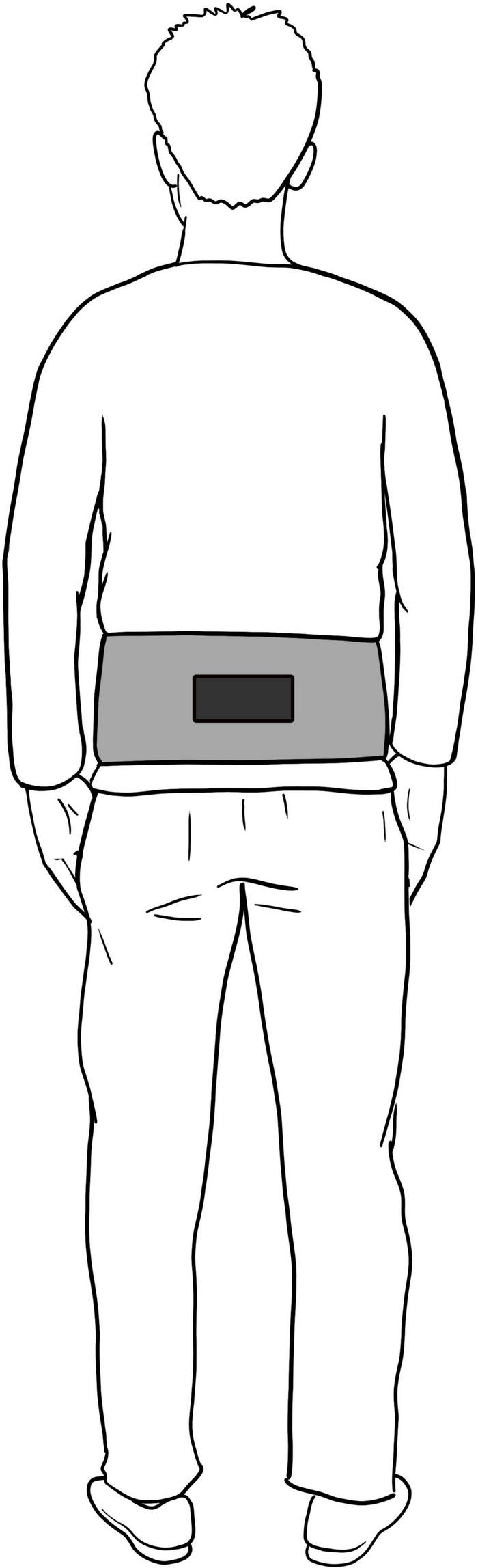
Set up of Fallskip device.

**FIGURE 2:**
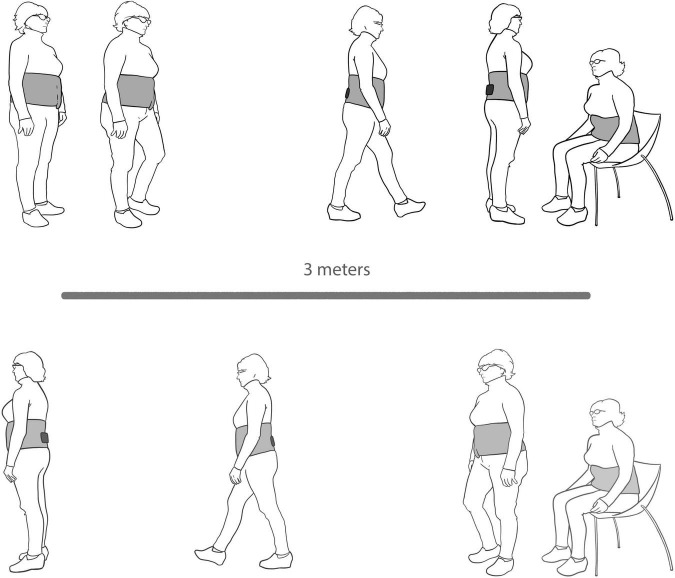
FallSkip protocol.

For the functional assessment, an inertial sensor embedded in the Android device FallSkip^®^ system (Biomechanical Institute of Valencia, València, Spain) (Serra-Añó et al., 2020) was used according to the protocol previously developed and validated by our group (Serra-Añó et al., 2019). The device is fixed with the height of L4–L5, approximately coinciding with the center of gravity (Figure 1). This functional assessment included five phases performed sequentially in a single recording (Figure 2):

- Phase 1. Standing still with arms alongside the body for 30 s.
- Phase 2. Walking straight ahead as fast and as safely as possible toward a chair 3 m away at the sound of an acoustic signal.
- Phase 3. Turning around and sitting down in a chair.
- Phase 4. Standing up from the chair.
- Phase 5. Walking back as fast and as safely as possible to the starting point.

Three variables were calculated for the static postural control phase: (i) Medial-lateral displacement (MLDisp) of the center of mass; (ii) Anterior-posterior displacement (APDisp) of the center of mass; and (iii) Displacement area (DispA) of the center of mass. Concerning gait analysis in phase 2, two variables were measured: (i) Vertical range (VRange) of the center of mass; (ii) Medial-lateral range (MLRange) of the center of mass (Serra-Añó et al., 2019).

Likewise, turning around and sitting down, and standing up from the chair were also monitored and two variables were calculated: (i) Turn-to-sit power (PTurnSit); (ii) Sit-to-stand power (PStand). Finally, two time-related variables were calculated: (i) Total time (TTime); (ii) Reaction time (RTime) before a sound stimulus (Serra-Añó et al., 2019).

### Statistics

Statistical data analysis was conducted using SPSS v26 (Inc., Chicago, IL, United States). The normality of the data distribution was analyzed with the Shapiro–Wilk test, and homoscedasticity was analyzed with Levene’s test.

For the inferential analysis, a between-subjects analysis of variance (one way- ANOVA) was performed to establish the PD level differences according to HY (I, II, and III) on the dependent variables (i.e., MLDisp, APDisp, DispA, VRange, MLRange, PTurnSit, PStand, TTime, and RTime).

## Results

### Participants

Eighty-seven individuals with PD were enrolled in this study, 30 women and 57 men (Table 1). The participants had a mean (SD) age of 69.03 (8.71) years, a mean weight of 74.41 (15.97) kg, and a mean height of 166.14 (8.31) cm. They were classified according to HY stages. For stages I, II, and III, 21, 30, and 36 volunteers were assessed, respectively.

**TABLE 1:** Demographic characteristics of the participants.

Outcome	All participants (*n* = 87)	HY-I (*n* = 21)	HY-II (*n* = 30)	HY-III (*n* = 36)
Age (years)	69.03 (8.71)	67.14 (8.20)*	66.10 (9.40)^‡^	72.58 (7.22)
Weight (kg)	74.41 (15.97)	72.36 (11.88)^†^	85.03 (18.72)^‡^	66.75 (9.80)
Height (m)	166.14 (8.31)	166.81 (6.92)	170.57 (7.34)^‡^	162.06 (7.97)
Sex (*n*, %)				
Women	30, 34.48	8, 38.10	5, 16.67	17, 47.22
Men	57, 65.52	13, 61.90	25, 93.33	19, 52.78

*HY-I, participant in stage I according to Hoehn and Yahr; HY-II, participant in stage II according to Hoehn and Yahr; HY-III, participant in stage III according to Hoehn and Yahr.*

*Data are expressed in mean (standard deviation).*

*^†^p < 0.05 between participants with level I and II.*

*p < 0.05 between participants with level I and III.

*^‡^p < 0.05 between participants with level II and III.*

### Effect of the Parkinson’s Disease Progression on Functional Mobility

Table 2 shows the comparison of the study variables in the three PD stages according to HY. There were significant differences between the two endpoint stages (i.e., I and III) in the three postural control variables (i.e., MLDisp, APDisp, and DispA), both PTurnSit and PStand, VRange during gait, and TTime. When the first consecutive stages were compared (i.e., I and II), only time and VRange significantly differed between stages, with a significant decrease in the vertical range and an increase in the time needed to complete the test. Finally, when stages II and III were compared, variables PTurnSit and PStand significantly decreased whilst TTime also increased in the most advanced stage.

**TABLE 2:** Differences between balance, gait, and risk of falls for different PD levels.

				Comparison HY-I and HY-II		Comparison HY-II and HY-III		Comparison HY-I and HY-III	
						
	HY-I (*n* = 21)	HY-II (*n* = 30)	HY-III (*n* = 36)	95% CI between	*d* Cohen	95% CI between	*d* Cohen	95% CI between	*d* Cohen
MLDisp	5.43 (2.65)	8.87 (8.35)	11.89 (8.81)	−8.61 to 1.74	−0.43	−7.52 to 1.47	−0.38	**−11.45 to −1.47**	**−0.81**
APDisp	18.44 (9.52)	21.02 (8.57)	27.07 (13.53)	−8.91 to 3.75	−0.22	−12.65 to 0.54	−0.52	**−16.02 to −1.24**	**−0.75**
DispA	294.42 (258.86)	717.18 (1,251.95)	1,100.22 (1,379.32)	−1,000.95 to 1,55.41	−0.35	−1,161.03 to 394.96	−0.32	**−1,382.48 to −229.13**	**−0.68**
VRange	32.4 (10.3)	26.23 (6.92)	22.38 (8.36)	**0.45 to 11.89**	**0.67**	−1.11 to 8.82	0.42	**4.51 to 15.54**	**1.09**
MLRange	44.13 (17.04)	43.27 (23.53)	49.67 (27.87)	−15.55 to 17.26	0.04	−20.65 to 7.85	−0.57	−21.37 to 10.29	−0.23
PTurnSit	111.67 (29.57)	96.93 (50.27)	65.33 (29.64)	−11.09 to 40.55	0.35	**9.17 to 54.03**	**0.75**	**21.42 to 71.25**	**1.09**
PStand	252.65 (97.75)	236.77 (74.02)	179.82 (76.6)	−39.31 to 71.09	0.18	**8.99 to 104.91**	**0.66**	**19.57 to 126.11**	**0.84**
TTime	11.83 (1.52)	14.35 (2.66)	16.77 (4.24)	**−3.94 to −1.09**	**−0.67**	**−4.48 to −0.36**	**−0.64**	**−6.83 to −3.05**	**−1.31**
RTime	1.03 (0.41)	1.24 (0.5)	1.24 (0.34)	−0.51 to 0.1	−0.49	−0.26 to 0.26	0.00	−0.47 to 0.05	−0.49

*Data are expressed as mean (standard deviation). HY-I, participant in stage I according to Hoehn and Yahr; HY-II, participant in stage II according to Hoehn and Yahr;*

*HY-III, participant in stage III according to Hoehn and Yahr. MLDisp, medial-lateral displacement; APDisp, anterior-posterior displacement; DispA, displacement area; VRange, vertical range; MLRange, medial-lateral range; PTurnSit, turn-to-sit power; PStand, sit-to-stand power; TTime, total time; RTime, reaction time.*

*Data in bold: significant differences between stages (p < 0.05).*

## Discussion

This study compared the functional status in patients with PD and its evolution throughout the stages of the disease (HY I, II, and III) using an easy-to-use inertial sensor embedded in an Android device with a single mobility test, including functional assessment of postural control, gait, turning and sitting and standing up from a chair (Serra-Añó et al., 2020).

Parkinson’s disease is a neurodegenerative disease whose progression can be slowed down using appropriate pharmacological and non-pharmacological interventions (Oliveira de Carvalho et al., 2018), such as physiotherapy and physical exercise focused on functional improvement, without ignoring the psychological assessment and treatment, that could interfere with physical therapies (Jiménez-Cebrián et al., 2021). Therefore, determining how the functional status evolves is of great interest to the management of this population.

Our results disclosed that the time required to complete the whole test (i.e., Ttime) significantly increased from one stage to the next, the proportion of change between stages I and II is 21.30%, and between II and III, 16.86%. Therefore, this is a useful variable to discriminate between stages. Previous studies had assessed the total time required to complete the Timed Up and Go (TUG) test and found that more advanced PD stages are associated with an increased time required to perform the test. However, these studies did not account for the HY stages and, therefore, our results are not entirely comparable (Schenkman et al., 2011; Helmy et al., 2022). Nonetheless, although this variable allows for differentiating the stages, clinical information regarding the physiological factors is missing. A previous review concluded that TUG has a limited ability to predict falls and should not be used alone to identify individuals at high risk of falls (Barry et al., 2014). Accordingly, other variables related to movement patterns are desirable to better understand the progression of the disease and better plan physical intervention to improve function (Brodie et al., 2014; Xu et al., 2018).

As described by HY, stages I and II show no balance dysfunction. In line with this, we obtained no differences between stages I and II (*p* > 0.05). However, displacement of the center of pressure significantly increased when comparing stage, stages I and III for medial-lateral, anterior-posterior displacement, and the area described by the center of pressure (i.e., MLDisp, APDisp, and DispA). In this regard, previous studies described the center of pressure trajectory as a useful clinical measure to identify postural control deficiencies (Rocchi et al., 2006; Błaszczyk et al., 2007).

Basal ganglia are one of the most affected structures in PD (i.e., *via* thalamic-cortical-spinal loops and *via* the brainstem pedunculopontine nucleus and the reticulospinal system) (Takakusaki et al., 2003). This impairment seems to be the principal cause of gait dysfunction and posture and balance deficits in this disease (Takakusaki et al., 2003).

Even though the increase in center of pressure displacements did not reach the level of significance, in the comparisons between both I-II and II-III, increases of the DispA of 143.59 and 53.41%, respectively, were obtained. Therefore, the lack of significance was probably due to the great amount of data dispersion, so further studies are needed to determine the possibility of postural control differences between PD stages including other possible confounder factors.

In terms of postural control, we explored two dynamic balance variables when walking, namely, the medial-lateral displacement and the vertical displacement of the center of pressure (i.e., MLRange and VRange). We did not obtain significant differences between stages in the MLRange, or even between stages I and III. This could be explained by the fact that this parameter already appeared altered in the first PD stage as compared to age-matched healthy people (Baltadjieva et al., 2006). Previous studies demonstrate that people with PD exhibit asymmetry in their dynamic center of mass trajectory during treadmill walking (Shin and Ahn, 2020).

On the other hand, VRange is a measure of the metabolic cost during gait (Cavagna and Margaria, 1966). Based on the inverted pendulum theory (Nguyen et al., 2017), whereby the stance leg acts as an inverted pendulum during gait, a certain extent of vertical displacement of the center of mass is needed, which would display the exchange between potential and kinetic energy during each stride. Our results showed that this vertical displacement was significantly reduced in stage II compared with stage I, as well as in stage III compared with stage I. However, there were no differences between stages II and III, which may indicate that the decrease of this displacement and, therefore, the increase of the metabolic cost during gait occur in the first stages. This could condition and explain the fatigue experienced in this population even in the early stages (Herlofson and Kluger, 2017).

Besides static and dynamic balance, we assessed two basic functional tasks, the sit-to-stand, and the reverse, plus turning the body (López-Pascual et al., 2018; Serra-Añó et al., 2020). We did not obtain significant differences between the first two stages but did, however, between I–III and II–III stages for the PStand. Concretely, the power with which the participants stood up from a chair was significantly lower as PD progressed. In this line, Mak and Hui-Chan (2005) concluded that people with PD take longer to complete the sit-to-stand task (including lower vertical and horizontal velocity) compared with a healthy control group. However, there is some controversy in this respect (Inkster and Eng, 2004; González Rojas et al., 2018), probably because studies do not differentiate between HY stages among their sample. Our results are consistent with the pathophysiologic mechanism of PD. The dopaminergic deficit in PD decreases the excitatory drive of the motor cortex, which directly affects the motor unit recruitment and produces muscle weakness (Lang and Lozano, 1998; David et al., 2011).

We also assessed turning around and sitting because it is more representative of daily life movements since the body needs to be suitably positioned relative to the chair before sitting. Our study findings were similar to those obtained for the sit-to-stand task, i.e., a significant decrease between stages II and III and between stages I and III. Our results are in line with those obtained in a recent study, in which turning and sitting were studied independently, thus, showing longer lapses of time to perform each of those tasks (Yahalom et al., 2020).

Finally, we explored the reaction time from an acoustic signal to gait initiation (i.e., RTime) due to the freezing of gait phenomenon, which typically occurs on initiating gait, likely caused by an inhibitory deficit in PD (Cohen et al., 2017). Overall, there were no significant differences between stages in reaction time. This was particularly evident for stages II and III, in which similar reaction times were obtained. Nevertheless, although not significant, the effect size of the difference between stages I and II was medium (d = 0.49), so this increase in the reaction time should be taken cautiously and analyzed. Freezing of gait appears at moderate to advanced stages of PD (Macht et al., 2007; Aktürk et al., 2021), hence, possibly explaining why the results for stages II and III are similar, as reaction time decreases in stage II and remains so thereafter throughout disease progression.

This study has some limitations. We did not include more advanced stages (IV and V), because the TUP test is not recommended for advanced stages. Besides, we did not register their mood status. Further, patients in our study were assessed in the “on” medication state. Therefore, our results cannot be extended beyond this condition. Finally, purposive sampling for the recruitment of the volunteers was used instead of simple randomization.

## Conclusion

Functionality in people with PD decreases throughout disease progression. Results showed that patients with advanced stage PD are slower and their static and dynamic balance is poorer. Moreover, the sit-to-stand power and the turning and sitting power decrease as PD progresses.

## Data Availability Statement

The raw data supporting the conclusions of this article will be made available by the authors, without undue reservation.

## Ethics Statement

The studies involving human participants were reviewed and approved by Ethics Committee of Universitat de València (H1517239006520). The patients/participants provided their written informed consent to participate in this study.

## Author Contributions

PS-A, JP-S, and JL-P: conception and design of the study, revising it critically, and final approval of the version to be submitted. NS-R, MI, EM-G, and MA-R: acquisition of data. PS-A, JL-P, JP-S, and SM-C: analysis and interpretation of data. PS-A, SM-C, and NS-R: drafting the manuscript. All authors have made substantial contributions and approved the submitted version.

## Conflict of Interest

The authors declare that the research was conducted in the absence of any commercial or financial relationships that could be construed as a potential conflict of interest. The handling editor, declared a shared affiliation with several of the authors SM-C, MI, EM-G, MA-R, NS-R, and PS-A, at the time of review.

## Publisher’s Note

All claims expressed in this article are solely those of the authors and do not necessarily represent those of their affiliated organizations, or those of the publisher, the editors and the reviewers. Any product that may be evaluated in this article, or claim that may be made by its manufacturer, is not guaranteed or endorsed by the publisher.
